# First Web Space Contracture

**Published:** 2014-08-21

**Authors:** Paul J. Therattil, Ramazi O. Datiashvili

**Affiliations:** Division of Plastic Surgery, Department of Surgery, Rutgers-New Jersey Medical School, Newark, NJ

**Keywords:** web space contracture, thumb reconstruction, 4 flap, web space deepening, hand trauma

**Figure:**
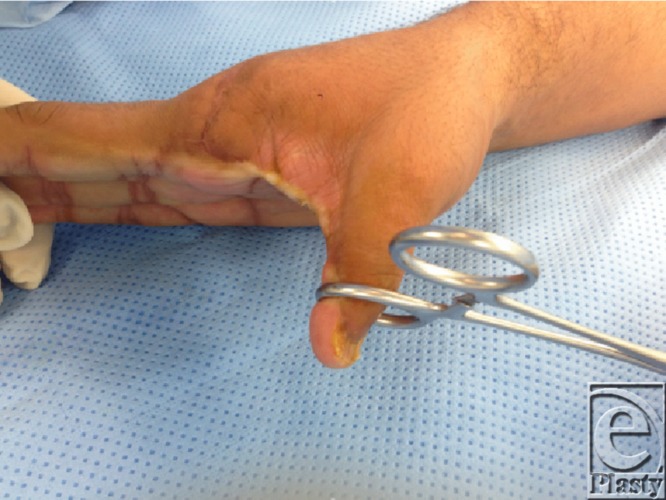


## DESCRIPTION

A 28-year-old man presents 5 months after a table saw crush injury to the thumb and the index finger of the right hand. Initially, he required thumb interphalangeal joint arthrodesis and ray amputation of the index finger. Postoperatively, the patient noted scarring and gradual contracture of the first web space causing pain with movement and significant restriction in the hand function.

## QUESTIONS

1. **How do first web space contractures affect overall hand function?**
2. **When is first web space deepening a viable reconstructive option?**
3. **What options are available for first web space reconstruction?**
4. **What local tissue rearrangements allow for the greatest deepening?**

## DISCUSSION

First web space contractures may result from traumatic injury, as with our patient, burns, or congenital hand malformations. These contractures have significant impact on hand function by restricting thumb opposition and abduction, and thus, impairing key pinch, pulp-to-pulp pinch, power grasp, chuck grip, and cradle grip.^1^ Considering the thumb accounts for 40% to 50% of the hand's function, first web contracture is a significant disability that should be addressed.

Injuries to the distal to the middle third of the proximal phalanx of the thumb require “phalangization,” or relative lengthening of the thumb. This can be accomplished by deepening the web space, as there is an inherent adduction contracture in these injuries. In order for deepening of the web space to be successful, the local skin should be relatively supple, the thumb metacarpal must be mobile, the intrinsic muscles should not be contracted, and at least half of the thumb length should be intact.^1^

Methods that have been used to release first web space contractures include skin grafting, Z-plasty variations (4-flap, 5-flap), V-Y plasty, first dorsal metacarpal artery flap, posterior interosseous flap,^2^ radial forearm flap, lateral arm flap, anterolateral thigh flap, and skeletal traction.^1^^,^^3^ While skin grafts are prone to contracture, dermal substitutes have been used recently with success in the treatment of web space contractures.^4^ In one case series by Askari et al,^5^ first web space contractures treated with release and application of dermal substitute retained 100% of postoperative range of motion and 90% to 100% of postoperative web space width with 1-year follow-up. Regardless, local flap options are the mainstay for web space release and deepening.

**Figure 1:**
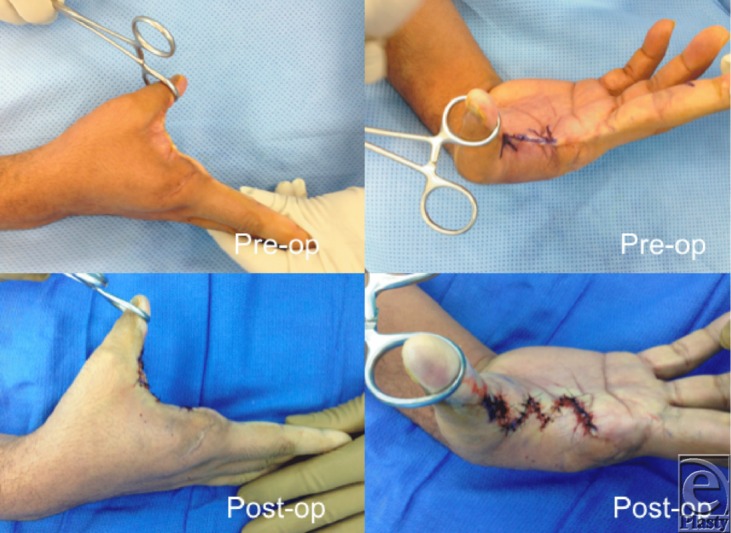
The right hand before- and after 4-flap Z-plasty for the first web space contracture.

In our patient, the skin surrounding the web space was of good quality and there was no obvious muscle contracture. Thus, we chose to perform a 90-degree 4-flap Z-plasty to release the contracture and deepen the web space (Fig. 1). There are several types of Z-plasty that can be used for web space deepening. Examination of plastic web space models demonstrated that a 90-degree 4-flap Z-plasty gave approximately 1.6 times greater deepening than a 5-flap Z-plasty; the 120-degree 4-flap Z plasty gave 2.0 times greater deepening than the 5-flap Z-plasty, and approximately 1.3 times greater deepening than the 90-degree 4-flap Z-plasty. This was tested and confirmed in porcine axillary webs. Although the 120-degree 4-flap Z-plasty gave greater deepening; it was harder to design and more difficult to close in this model.^6^

**Figure 2:**
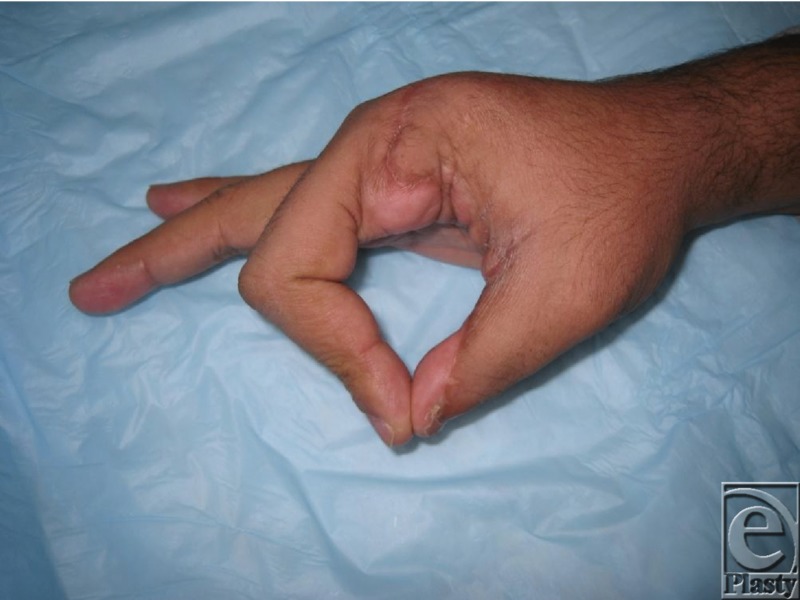
Postoperative function of opposition.

Successful postoperative care is equally important to maintain web space release, and for our patient included placement in a thumb spica splint to maintain the web space during the interval healing period. The patient was able to achieve excellent thumb opposition and abduction, which were his initial deficiencies, as well as improved overall hand function (Fig. 2). It can be seen from this case that local tissue transfer is an excellent option for web space deepening, although choosing the optimal configuration is key to achieving the best release.
